# Clinico Psycho Social Conference: A Novel Learning Experience for Postgraduate Students of Community Medicine

**DOI:** 10.4103/0970-0218.42064

**Published:** 2008-07

**Authors:** Dinesh Kumar, Amarjeet Singh

**Affiliations:** Department of Community Medicine, School of Public Health, Postgraduate Institute of Medical Education and Research (PGIMER), Chandigarh, India

## Introduction

The School of Public Health (SPH), Postgraduate Institute of Medical Education and Research (PGIMER), has initiated a Clinico Psycho Social Conference (CPSC) for its postgraduate students. Focus is on the social aspects of the disease. Present case attempts to demonstrate the potential of CPSC as a training method.

The index case was a female, who got married at 35 years of age to a graduate farmer from a joint Jat family of Punjab. She conceived within 2 months of marriage. A private practitioner referred her to government hospital. Her husband accompanied her to the hospital, where she was confirmed HIV positive. She was then referred to PGIMER.

The patient was in a state of shock, initially, when she knew about her HIV status, as she said, *“Eh bimari mainu kithon lag gai, kyun lag gai? ? ?”* (from where and why did I get this disease?). Her husband supported her in this phase of crisis as she told, *“Pati ne keha ki bade hospital vich dikha ke dekhiye”* (My husband suggested that we should take a chance and consult a bigger hospital.). Her main concern seemed to be the survival of the fetus. She said, *“Sayad bacha bach jave, kafi muridan ton mangaya hai”* (May be our child survives; the conception was a result of lots of praying to the God).

She was counseled in PGIMER on various issues such as immunization, breast feeding, etc. She regularly took Ziduvudine. She told, *“Davai Khani hai, CD4 jaida hai ate AIDS hon vich time lagda hai”* (The doctor told me that my CD4 count was high; it takes time for the development of AIDS, so I have to take the medicine).

She did not tell her in-laws about her HIV status. Her mother, sister and brother accompanied her to the hospital. She gave birth to a 4-kg male baby through caesarian. About her plans, she told that *“asi hun bachha karna hi nahin”* (We do not want another child). Her husband was reported to be HIV negative.

## Application of Theoretical Models of Behavior to the Present Case

Social cognition models explain determinants of health behavior in terms of action taken to prevent and detect disease, e.g. visiting doctors and complying with advice.

### Stress and coping

First, we tried to explain the coping strategies adopted by the index case to deal with a stressful situation i.e. HIV positive status. According to the Lazarus theory of stress and coping,(1) patients respond to stress in the following stages:

- **Stress:** When patients learn about the serious nature of their disease, they experience stress.
- **Primary appraisal:** They evaluate whether there is a demand on their resources, i.e. threat of harm, loss or challenge.
- **Secondary appraisal:** They evaluate their ability to cope with the demand using personal or environmental resources.
- **Coping strategies:** They deal with the demand through problem-focused strategies and emotional strategies.
- **Outcome:** They modify their health behavior in order to fulfil their social role.

All these stages of dealing with the disease-related stress were witnessed in the present case. Stress due to the nature of the disease (a potentially fatal one) was visible in the responses given by the patient. The patient found her situation stressful, negative, uncontrollable, threatening and challenging. Thereafter, she evaluated her resources. Health system support was available to her in the form of the initial diagnosis by the private practitioner followed by appropriate referral to a government hospital, services available at the apex institute (counseling) and facilities available through the State AIDS Control Society (SACS), e.g. free antiretroviral therapy, knowledge gained from health staff, along with social support of husband and her parents. As a response to the problem faced by them, the patient and her family complied with the referral as well as the therapy recommended. Adequate emotional support of parents and the husband of the index case was observed. Eventually, the index case gave birth to a healthy baby. The couple exhibited responsible parenthood behavior e.g. decision of not bearing child in future.

### Health belief model

According to health belief model (HBM),(2) patients must hold the following beliefs in order to able to change their behavior, which depend upon their socio-demographic characteristics, knowledge and attitudes.

- **Perceived susceptibility:** They perceive themselves susceptible to the disease.
- **Perceived seriousness:** They realize disease as serious enough to cause harm.
- **Perceived benefits:** They perceive the proposed health action as beneficial.
- **Perceived barriers:** They realize that benefits outweigh any disadvantage because of the proposed action.
- **Cues to action and self-efficacy:** They recognize that these barriers can be overcome with the help of supporting environment.

HBM could also be visualized in the behavior of the index case. In the beginning when the diagnosis was revealed, the patient wondered *“Ki Mainu AIDS ho gai hai?”* (Whether I have contracted AIDS?). The patient recognized the severity of the disease i.e. HIV/AIDS is a potentially fatal disease and related it to a death in her village *“15 saal pehale sadhe pind vich isi bimari ton ek bande di maut ho gai sigi, lagdha hai ki meri vi Hun jindgi khatam hai”* (A patient died in our village of this disease. Now I felt I would also die.). Here, the perceived seriousness of the disease was affected by her previous knowledge. With the available government publicity, the patient knew that the survival of the baby would be the main advantage of treatment. Apparently, the messages through media about the therapy had affected her evaluation of the benefits. The patient weighed the pros and cons of various proposed actions and realized that the survival of her child will outweigh any stigmatization due to her being on treatment for HIV. This was due to the prevailing value system in the society about the male child. Ultimately, with the family support, the patient was encouraged to overcome the fear of severity of AIDS and the stigma.

### Health action model

As per health action model (HAM),(3) patients' intention to undertake any given health action is influenced by their beliefs, motivational factors and normative pressures. Supportive environment (social, economic and physical), knowledge and life skills facilitate translation of intention into practice.

- **Belief system:** Before consultation, patients must aware of the disease and must believe in its severity. They must justify their visit to doctors. Such beliefs are affected by their existing beliefs regarding the disease (risk factors, its seriousness and perceived susceptibility).
- **Normative system:** Despite our own beliefs and personal preferences, our intentions are influenced by what other people think about the issue, i.e. general social norms and the more direct impact of significant others (spouse, family or peer group). General social norms conveyed via mass media exert less pressure than direct personal, interpersonal interaction/experiences of our primary group.
- **Motivational system:** Individuals' values form an important part of the motivation system. Each value will generate a variety of attitudes. The motivational system recognizes that certain basic and powerful influences and “drives” that are largely inherited and “instinctive” species-specific motivational factors, e.g. hunger, sex and pain, can override the socially acquired values and attitudes to influence the decision making. In addition, the presence of certain emotional states may signify the existence of motivational factors derived from drives e.g. guilt and anxiety.

In this case also, patient's self-concept, belief system, motivation system and normative system facilitated her going to PGIMER. Systems support, availability of health facilities (counseling and free drugs) and private practitioner helped the patient to come to PGIMER. For women in India, going to hospitals is fraught with many barriers. In this case, the support by her husband (who escorted her to the hospital) was the critical factor in the initiation of the treatment. Supportive messages through mass media might also have helped in shaping his attitude as favorable towards his HIV positive wife.

The chain of treatment-seeking behavior started when the patient was diagnosed as HIV positive, as she said, *“Test pakka ho gaya hai, AIDS tah hai”* (The test has confirmed that I have AIDS.). She was also aware of the potential fatal nature of the disease. The hope of the survival of the fetus in the womb of an HIV positive mother led to appropriate treatment-seeking behavior. This reflected her desire to fulfil the social role as a mother. In Indian context, giving birth is considered as a duty and norm for women because of our belief in rebirth and need of a son for salvation. Inability to do this stigmatizes women. The concern of the parents for survival of a valued child overcomes any potential stigma resulting from treatment.

Thus, the present example shows that how an individual's intention to seek treatment can be translated into practice, when a variety of facilitating factors are in place. This example also demonstrates through application of HAM that the prevailing atmosphere must be favorable if the healthy choice is to be made an easy choice.

Our experience revealed that the use of CPSC for postgraduate medical education in Community Medicine helps in better illustration and understanding treatment-seeking behavior of patients. Such approach should also be aimed among other public health institutions and medical colleges so that all of us can exchange notes and improve it further.
